# VEXAS syndrome: cutaneous manifestations and *UBA1* gene variants in the diagnosis of a rare autoinflammatory syndrome – Case report^☆^

**DOI:** 10.1016/j.abd.2025.501233

**Published:** 2025-11-03

**Authors:** Isabel Crivelatti, Shirley Massimo de Souza, Oscar Cardoso Dimatos, Gabriella Di Giunta Funchal, Andressa Miozzo Soares, Amanda Amaro Pereira

**Affiliations:** Hospital Universitário Polydoro Ernani de São Thiago, Universidade Federal de Santa Catarina, Florianópolis, SC, Brazil

*Dear Editor,*

VEXAS syndrome (VS) is a rare, recently described autoinflammatory disease that primarily affects men over 50 years of age and is caused by somatic mutations in the *UBA1* gene, located on the X chromosome.^1^, ^2^ The clinical presentation includes constitutional symptoms (fever, night sweats, fatigue, weight loss) and hematological, musculoskeletal, cardiopulmonary, and cutaneous manifestations (Fig. 1).^2^, ^3^, ^4^, ^5^, ^6^

**Fig. 1:**
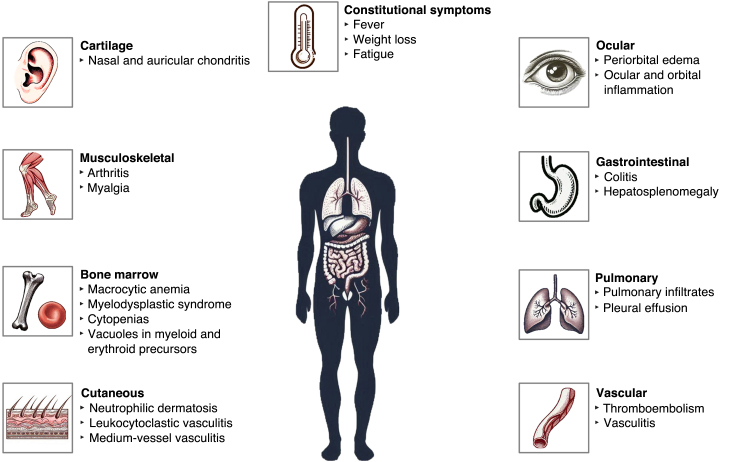
Clinical manifestations of VEXAS syndrome. Source: The authors, 2025. Created with Microsoft Designer 2025.

Skin involvement occurs in 80%–100% of cases and is characterized by erythematous and/or violaceous papules and plaques, sometimes edematous, predominantly on the back, upper limbs, neck, and face. Erythematous to purpuric papules and plaques are mainly observed on the lower limbs.^7^ The cutaneous condition is often initially diagnosed as Sweet's syndrome or vasculitis based on the histopathological findings of neutrophilic dermatosis and leukocytoclastic vasculitis, respectively.^1^ Although there are no established diagnostic criteria for VS, bone marrow evaluation is essential to identify cytoplasmic vacuoles in myeloid and erythroid precursors. However, for a definitive diagnosis, genetic confirmation of mutations in the *UBA1* gene is necessary.^8^

Treatment is still uncertain, with glucocorticoids being the most useful medications.^8^ The prognosis is poor, with a 5-year mortality rate of 30%–40%.^7^ However, bone marrow transplantation and gene-editing therapies appear promising, according to recent studies.^9^ The objective of this case report was to highlight the cutaneous manifestations in VS and to reinforce the importance of genetic testing in patient prognosis and in understanding VS.

A 64-year-old man presented to the emergency room due to weight loss of 16 kg in the previous six months, blood in the stool for five months, anemia for four months, a history of fatigue for one month, and fever above 38 °C for three days. He also reported the appearance of asymptomatic skin lesions for one week. Physical examination revealed erythematous-violaceous plaques on the trunk, back, upper limbs, palms, soles, and face, and violaceous macules and papules on the lower limbs, which did not disappear with digital pressure (Fig. 2A- and B). Laboratory tests revealed elevated serum C-reactive protein (CRP) of 152 mg/L, macrocytic anemia with a hemoglobin of 6.2 g/dL and a mean corpuscular volume of 116.5 fL, neutrophilia of 14,400 μL, 23% of which were rods (3,319 μL), and thrombocytopenia of 53,000 μL.

**Fig. 2:**
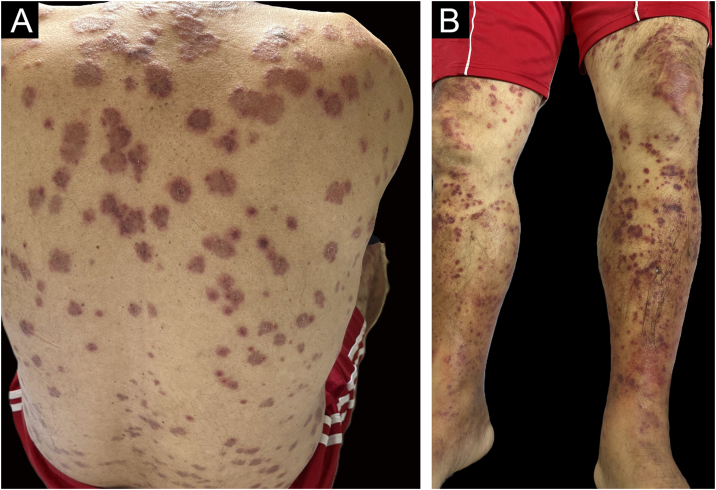
Cutaneous manifestations. (A) Erythematous-violaceous papules and plaques, with edematous edges, some with a tendency to coalesce, on the back. (B) Palpable purpura on the lower limbs.

Biopsies were performed of skin lesions on the trunk, consistent with Sweet's syndrome, and on the left lower limb, consistent with leukocytoclastic vasculitis (Fig. 3). Direct immunofluorescence of the lesions on the lower limb showed no immune deposits.

**Fig. 3:**
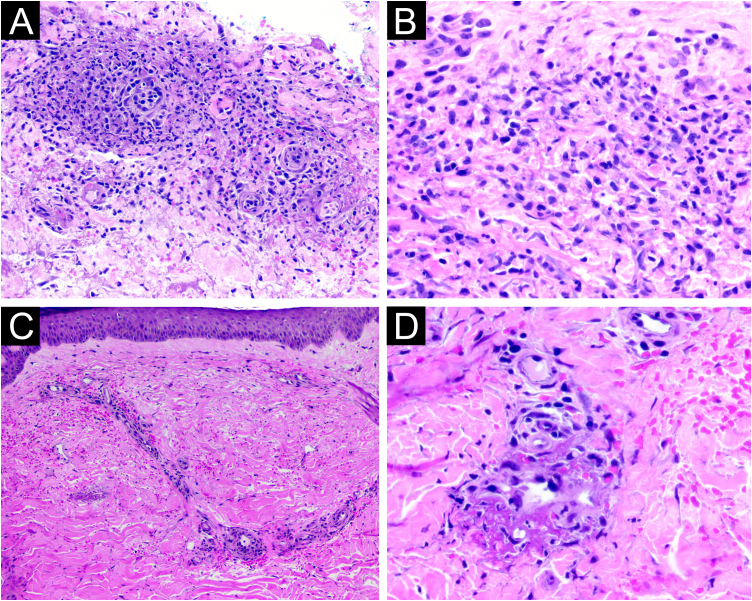
Histopathological findings. Biopsy of a lesion on the trunk showed mono- and polymorphonuclear infiltrate, with particulate neutrophils and extravasated red blood cells, without signs of vasculitis (A, Hematoxylin & eosin, ×200; B, Hematoxylin & eosin, ×400). Biopsy of a lesion on the lower limb showed extravasated red blood cells, mixed perivascular inflammatory infiltrate with leukocytoclasia, and the presence of fibrin thrombi in the capillary wall (C, Hematoxylin & eosin, ×100; D, Hematoxylin & eosin, ×400).

For initial management, prednisone was prescribed at a dose of 1 mg/kg/day, with significant improvement of the lesions 48 hours after starting corticosteroid therapy. Antibiotic therapy was also administered for fever associated with leukocytosis with a left shift in the leukogram, and erythropoietin was administered for anemia.

The bone marrow biopsy revealed findings suggestive of myelodysplastic syndrome, and a bone marrow biopsy revealed hypercellularity with myelodysplastic/myeloproliferative abnormalities associated with megakaryocytosis and grade 1 myelofibrosis. Based on these findings, VS was hypothesized. Genetic sequencing of the patient’s bone marrow specimens revealed the presence of the c.1741 + 15G>A mutation in the *UBA1* gene (Fig. 4).

**Fig. 4:**
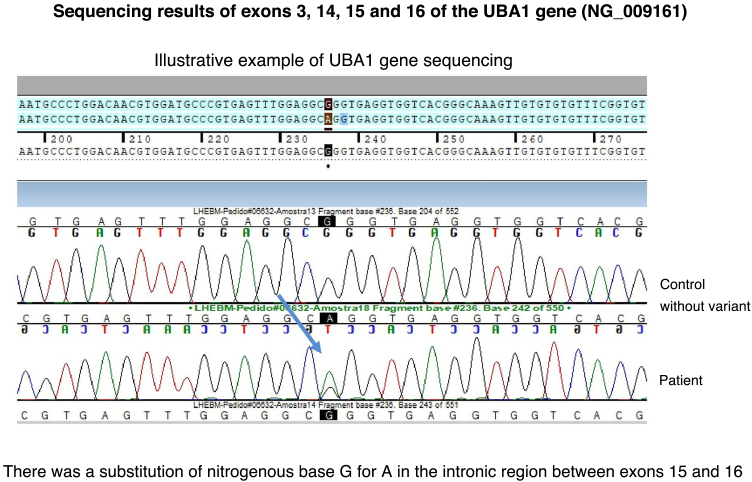
Genetic testing of the *UBA1* gene with DNA sequencing using the Sanger method of exons 3, 14, 15 and 16. Mutation identified between exons 15 and 16, with the nitrogenous base G being replaced by A.

Skin changes, present in most patients with VS, are usually the first manifestation of the syndrome, reinforcing the essential role of the dermatologist in early identification of the disease. In a case of Sweet's syndrome associated with vasculitis in the lower limbs and constitutional symptoms, in addition to the involvement of other systems, especially hematological ones, in men over 50 years of age, VS should be considered as a differential diagnosis.

Early and timely recognition of suspected cases leads to assertiveness in ordering genetic testing. The identification of new pathogenic somatic variants in the *UBA1* gene allows targeted genetic evaluation, making it faster and more effective. There are possibly still numerous undescribed pathogenic variants related to VS. Genetic sequencing of this patient identified a variant of uncertain significance, as it is not among those already described to date (Fig. 4).^10^ Therefore, it is presumed that this is yet another unrecognized VS-related variant.

Raising awareness of VS among dermatologists is essential to facilitate early diagnosis. New pathogenic variants in the *UBA1* gene identified in VS should be documented for a better understanding of the disease and more efficient genetic evaluation, aiming to mitigate the high morbidity and mortality.

## ORCID IDs

Shirley Massimo de Souza: 0009-0003-7561-6184; Oscar Cardoso Dimatos: 0000-0002-2201-0387; Gabriella Di Giunta Funchal: 0000-0001-8383-2918; Andressa Miozzo Soares: 0000-0002-5823-2563; Amanda Amaro Pereira: 0000-0002-8735-7584

## Authors’ contributions

Isabel Crivelatti: Design and planning of the study; drafting and editing of the manuscript or critical review of important intellectual content.

Shirley Massimo de Souza: Collection, analysis, and interpretation of data; effective participation in research orientation.

Oscar Cardoso Dimatos: Drafting and editing of the manuscript or critical review of important intellectual content; effective participation in research orientation.

Gabriella Di Giunta Funchal: Collection, or analysis and interpretation of data; critical review of the literature.

Andressa Miozzo Soares: Drafting and editing of the manuscript or critical review of important intellectual content; intellectual participation in the propaedeutic and/or therapeutic conduct of the studied cases.

Amanda Amaro Pereira: Data collection, or analysis and interpretation of data; intellectual participation in the propaedeutic and/or therapeutic conduct of the studied cases.

## Financial support

This research did not receive any specific financial support from public, private, or non-profit funding agencies.

## Availability of research data

Not applicable.

## Conflicts of interest

None declared.
